# Organoid Transplantation Can Improve Reproductive Prognosis by Promoting Endometrial Repair in Mice

**DOI:** 10.7150/ijbs.69410

**Published:** 2022-03-21

**Authors:** Huixing Zhang, Dabao Xu, Yan Li, Jianqiang Lan, Yu Zhu, Jia Cao, Mingyue Hu, Juanjuan Yuan, He Jin, Gang Li, Dan Liu

**Affiliations:** 1 Department of Gynecology, General Hospital of Ningxia Medical University, Yinchuan, Ningxia, China; 2 College of Clinical Medicine, Ningxia Medical University, Yinchuan, Ningxia, China; 3 Nanfang Hospital, Southern Medical University, Guangzhou, China; 4 Key Laboratory of Ministry of Education for Fertility Preservation and Maintenance, Ningxia Medical University, Yinchuan, Ningxia, China; 5 Guangdong Research Center of Organoid Engineering and Technology, Guangzhou, Guangdong, China; 6 Department of Gynecology, Third Xiangya Hospital of Central South University, Changsha, Hunan, China

**Keywords:** Intrauterine adhesion (IUA), Three-dimensional culture, Endometrial organoids ansplantation, Endometrial regeneration

## Abstract

Objective: Intrauterine adhesion (IUA) is one of the major causes of refractory secondary infertility, especially in regions and countries with high abortion rates. In this study, we used the mouse IUA model to evaluate the feasibility of the organoids, a 3D cell structure derived from endometrial tissue, as grafts for the treatment of post-traumatic endometrial regeneration disorders. Methods: The isolated and cultured endometrial organoid was transplanted into the model IUA uterus by the hydrogel scaffold method. Results: The cultured endometrial organoids were transplanted into the basal layer of the damaged endometrium for 28 days. They were completely implanted and grew normally. They not only reconstructed the structural integrity of the endometrial epithelium but also realized the functional repair of the endometrium through differentiation cultures and secretory functions. Conclusion: For severe IUA, this method may be better than stem cell transplantation. These findings provide useful insights into the use of endometrial organoid regeneration in the treatment of injury repair.

## Introduction

Endometrial trauma is the major cause of Asherman syndrome or Intrauterine adhesion (IUA), which may lead to a series of complications such as abnormal menstrual bleeding, recurrent abortion and secondary infertility^1^ . The stem cell transplantation that aimed to repair the endometrial tissue damage by promoting the endometrial regeneration has been proved as an efficient treatment method against IUA in both basic research and clinical trials^2^-^4^. However, the low survival rate of stem cells after transplantation and the unmanageable differentiation status, which has the carcinogenic potential^5^, limited its large-scaled clinical application. Therefore, a new technique, which could meet the quality demands of the stem cell sources and the transplantation safety requirement is highly required.

The etiology of IUA is usually related to the injury of endometrial basal layer, especially after intrauterine surgery such as hysteroscopic surgery and induced abortion. At the same time, a variety of adjuvant treatment measures, such as IUD placement, balloon placement and estrogen treatment, are expected to improve the surgical efficacy and reduce the incidence of postoperative adhesion. However, the improvement of reproductive function hovered at 40-50% over the past decade^6^-^8^. Based on the experience of two months' after operation and B-ultrasound monitoring of endometrial thickness, we found that it is very difficult for patients to conceive, including the use of assisted reproductive technology, regardless of whether the second look hyperscopic detection indicates re adhesion and if the endometrial thickness in the late proliferative stage monitored by B-ultrasound is less than 7mm, these patients have poor endometrial blood flow and poor response to estrogen, which means that functional regeneration is not achieved. The treatment of endometrial regeneration by stem cells seems to be a new hope. Ramyar Azizi et al. have shown that stem cell transplantation has a positive effect on IUA treatment^2^. However, it also has its defects in application, such as the low survival rate of stem cells after stem cell suspension transplantation and the need to control the abnormal proliferation and differentiation of stem cells, and there are carcinogenic (IPSC) derivatives after differentiation. A new emerged three-dimensional (3D) cell structure, organoids are derived from stem cells, but the results of *in vitro* culture and amplification can faithfully reproduce the characteristics of primary organ, which overcomes this obstacle^9^-^11^. Organoid is an 3D cellular structure, could derived from either pluripotent (embryonic or induced) or adult stem cells from various organs. It is capable to form an 3D* ex-vivo* structure that mimic the key structural and functional features of desired organs under specific culture conditions^12^ and therefore has been widely used to study the developmental biology and tissue regeneration mechanism^13^. Unlike the traditional two-dimensional primary stem cell culture, organoids show higher amplification ability and could overcome the limited availability and scalability of autologous tissue and reshape the characteristics of the damaged tissue^14^. Up to date, many tissue-specific organoids culture models, including endometrial organoid model, have been established^15^-^17^. Endometrial organoid (EMO) derived from tissue stem cell has been shown could expand long-term and are genetically stable and differentiate following treatment with reproductive hormones^18^. At present, organoid as potential tissue repair tool has been studied for diverse tissues, such as brain^13^, lung^19^, ^20^, kidney^21^, liver^22^, ^23^. However, the potential tissue repair capability of endometrial organoids was less investigated. In this study, we have successfully cultured mature endometrial organoids and established a murine IUA model. Using this model, we have investigated the tissue repair effect of endometrial organoids by transplanting the organoids into IUA mice. Our results shown that endometrial organoids promote the endometrial tissue repair in the IUA mice and provide preliminarily evidence that endometrial organoids could have high guiding significance for endometrial tissue regeneration.

## Materials and Methods

### Subjects

This study was conducted in strict accordance with the recommendations of the National Institutes of Health guidelines for the care and use of laboratory animals. The experimental protocol and animal treatment procedure were approved by the Ethics Committee of the General Hospital of Ningxia Medical University. Female C57 were purchased from the experimental animal center of Ningxia Medical University. All animals were maintained in accordance with the public health service policy “Humane Care and Use of Experimental Animals,” the U.S. Department of Health's “Guidelines for Humane Care and Use of Experimental Animals,” and the provisions of the U.S. Department of Agriculture.

### Animal Experiment Design

Female C57 (6-8 weeks old, 150-200g) were purchased from the experimental animal center of Ningxia Medical University. The feeding temperature was 20-26 ℃; the humidity was 50-60%; and the light/dark cycle was 12h. Every morning from 8:00 to 10:00, vaginal smears were taken to check the estrus. Female mice were randomly divided into 3 groups after 2 estrous cycles: control group (n = 12), after abdominal cavity opening, uterine horn was exposed to air for 30 minutes and then sutured; IUA model group (n = 12), the uterine horn was only mechanical injured without any treatment (the uterus injury status was checked at 7, 14, 21, and 28 days after the operation); Transplantation group: one week after the establishment of the IUA model, the organoid suspension was prepared and transplanted into each uterine cavity (the uterus injury status was checked at 7, 14, 21, and 28 days after the operation).

### Isolation And Culture of Mouse Endometrial Organoids (EMOs)

After sacrifice the mouse, the abdominal cavity was opened and the exposed uterus was taken out, transferred into the culture dish. Subsequently, the uterus was opened longitudinally and dissected into small fragments, and enzymatically digested in 5 ml enzyme digestion solution (0.4 mg/ml collagenase v [sigma], 1.25 U/ml dispase II [sigma]) with gentle shaking at 37°C for 40 - 50 minutes. 10 ml of neutralizing medium (DMEM/F12, add 1% antibiotic) was added to stop the digestion. The solution mix was centrifuged and the pellet was washed three times with PBS (Phosphate Buffered Saline, without Ca2+/Mg2+). After wash, the solution mix was filtered through a 100 µM cell filter and the flow-through was collected and centrifuged at 1200 R/g for 3 minutes. Next, the supernatant was removed and the pellet was resuspended in Matrigel (Corning) at a volume of 1:1.5-1:2 and 20uL drops of Matrigel-cell suspension were transferred into a preheated 24 well plate. After solidification, culture medium was added and the endometrial organoids were cultured in the 5% CO2 incubator at 37℃. The culture medium was refreshed every 2-3 days. Cultures were passaged from 1:2 to 1:3 every 7-10 days.

### Hormone Therapy of Endometrial Organoids (EMOs)

To determine the sensitivity of organoids to hormones, 3-5 x 10^6^ cells/matrix gel droplets were plated in 12 well plates (3 droplets per well) and cultured for 4 days. Subsequently, organoids were treated with 10 nm estradiol (E2, sigma) for 2 days and followed by 6 days combi-treatment with 10 nm E2, 10 um P4 and 1 U cAMP (2'—dibutyl adenosine, 3', 5'—cyclic sodium phosphate; sigma) (E2 + P4 + cAMP) . The morphology change of the organoids was determined by taking photos under microscope and histological assessment.

### Establishment of IUA Model and Transplantation of EMOs

The IUA model was established through the mechanical damage method. Briefly, mice were anesthetized by intraperitoneal injection of 5% pentobarbital sodium (5mg/kg). After that, the abdominal cavity was opened with sterile scissor to expose the uterine horn. Subsequently, two 0.1 cm incisions at the bottom of the uterus were made and n two-thirds of the uterus was scraped with a 5 mm uterine curette until the uterine wall was pale and rough, both uterine horns were treated equally. After that, the uterus and abdominal cavity were cleaned and the wound was sutured and disinfected with iodophor solution.

EMO transplantation was conducted one week after the establishment of IUA model. Briefly, after anesthetized, mice abdominal cavity was opened with sterile scissor to expose the uterine horn. Next, 40 ul of 1 x 10^7^ mature organoids resuspend in 15% hydrogel were transplanted into each uterine cavity and the uterine cavity was ligated on both sides. After that, the uterus and abdominal cavity were cleaned and the wound was sutured and disinfected with iodophor solution.

### Immunohistochemistry and Periodate Schiff Staining

To collect organoids, we removed the organoid from matrix gel and immobilized it overnight in paraformaldehyde (PFA, 4% in PBS) at 4°C with paraffin embedded.Monoclonal antibodies against CK 7, Ki67 and EpCAM were added at a working concentration of 1:1 000. Immuno-stained sections were counter stained with hematoxylin. If necessary, antigen uncovering was performed in a sodium citrate buffer (10 mm, pH 6) at 95°C for 30 minutes.

Periodic acid—Schiff (PAS) staining was able to show the presence of mucin and was conducted following the manufacturer's instructions (sigma Aldrich). In short, sections were dewaxed and continuously cultured in periodate solution and Schiff reagent. The sections were counterstained with hematoxylin and fixed before dehydration.

### Electron Microscope Analysis

Mice endometrioid organoids were fixed with 5% glutaraldehyde at 4℃ for 4 hours and cut into 1mm × 1mm × 1mm fragments. After that, sections were incubated in 1% cesium tetrachloride for 2 hours, followed by routinely dehydration, tissue embedding and ultra-thin section. After 2% uranium acetate and lead citrate staining, the ultra-thin sliced sections were projected under transmission electron microscopy and photos were taken.

### RNA Extraction and Quantitative Polymerase Chain Reaction

Total RNA was extracted using a Trizol reagent (Invitrogen, Carlsbad, California, USA) and, subsequently, reverse transcribed into cDNA using the all-in-one first-strand cDNA synthesis kit according to the manufacture (Genecopoeia, Guangzhou, Guangdong, China). Real-time PCR was conducted using the real-time PCR kit purchased from Genecopoeia with the CFX 96 Real-time system (BioRad, Hercules, CA USA). All used primers were listed synthesized by Genecopoeia. The targeted gene expression was relatively quantified using the comparative threshold circulation (CT) method. This value was used to plot gene expression with the equation 2 ΔΔ CT and the final data for the target samples were normalized against the internal control GAPDH.

### Statistical Analysis

Statistical analysis was performed using SPSS 20.0 (IBM, USA) and GraphPad Prism version 6.0 (USA). The data are presented as means ± SD. Statistical differences between two groups was determined using T-test and statistical differences among multiple groups were determined by one-way analysis of variance (ANOVA). P < 0.05 was considered to be statistically significant.

## Results

### Established Mice Endometrial Organoids (EMOs) Recapitulate Morphology Features of Endometrial Tissue* in vivo*

**Figure 1:**
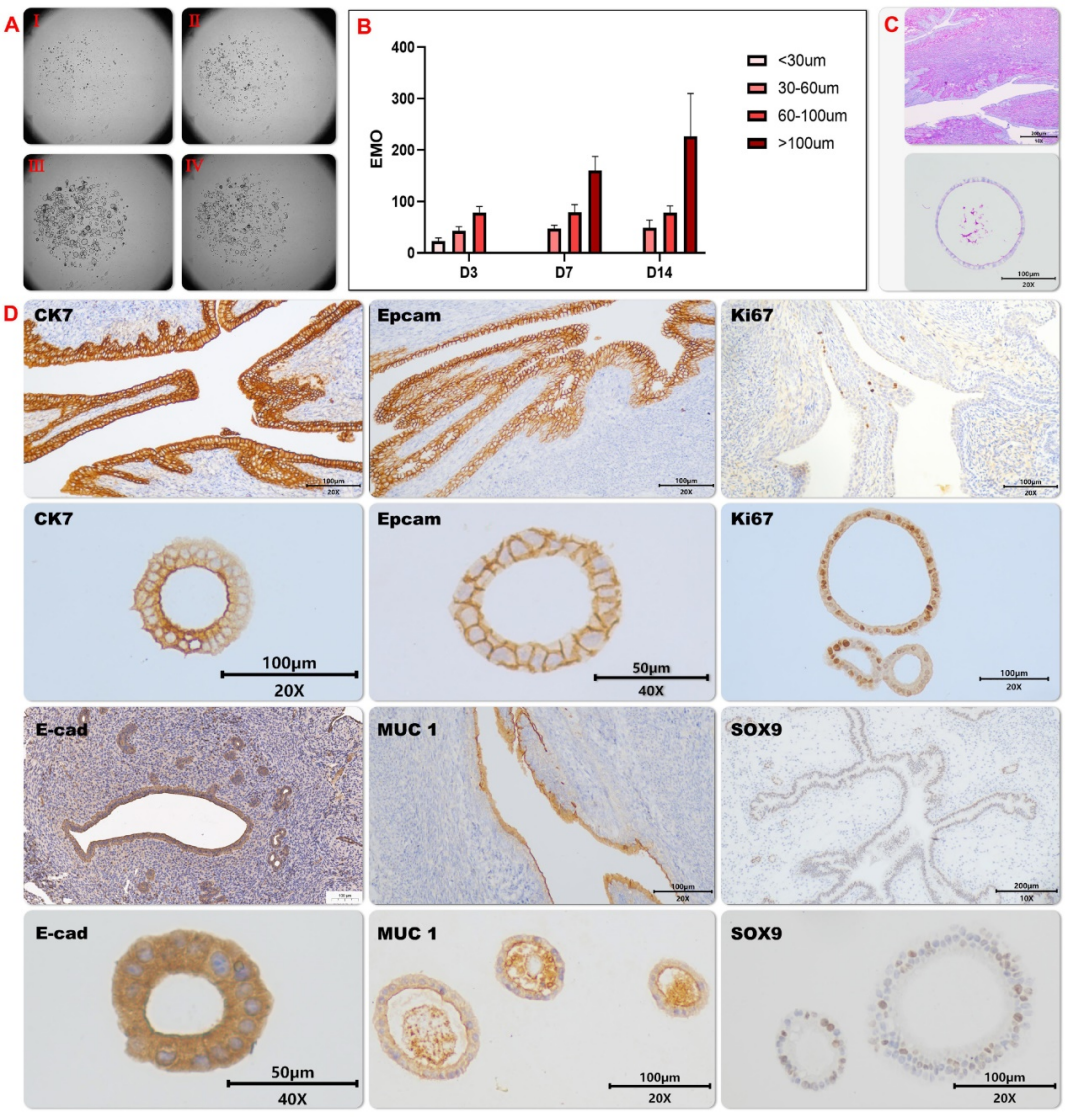
** Cultivation and characterization of mice endometrial organoids.** The growth of endometrial organoids on different time points were captured under the Light microscope (Fig. 1A) and the average diameter of endometrial organoids on indicated days was illustrated by the bar graph (EMOs) (Fig. 1B); The representative pictures of Periodic acid—Schiff (PAS) staining of endometrial organoids and its parental tissue were shown (Fig. 1C). The representative pictures of immunohistochemical staining for cytokeratin-7 (CK7), E-cadherin (E-cad), EpCAM, MUC-1 and SOX9 of endometrial organoid and its parental tissue were shown (Fig. 1D ) .

To explore the tissue repair capability of endometrial organoids (EMOs), we have established a proper culture condition for mice endometrioid organoids, which promote cellular differentiations and self-renewals simultaneously. To generate endometrial organoids, we used tissue isolates enriched for epithelial cells, and allowed these to self-organize within Matrigel droplets with the basal medium that supports development of other mice tissue organoids^14^, containing 80 ng/ml EGF; 150 ng/ml Noggin; 500 ng/ml R-spondin 1; 150 ng/ml Wnt3a; 5 ng/ml FGF-10; 30 ng/ml KIAA1199; 100 nM CHIR99021; 8μM A83-01, 8μM RKI-1477; B27, 1X; N-acetylcysteine, 1.25mM; Nicotinamide, 10 mM; HGF,50ng/ml; N2, 1X 100 ug/ml primary cell antibiotic. Using this culture condition, endometrial stem cell quickly started to reorganize into cell clusters and began to expand (Figure 1A and B). The generated organoids were composed of columnar epithelium cells with cystic or vacuoles appearances, and secretions were observed in the lumen of organoids (Figure 1C). This observation indicates that EMO may be composed of glandular epithelial cells with secretory functions.

**Figure 2:**
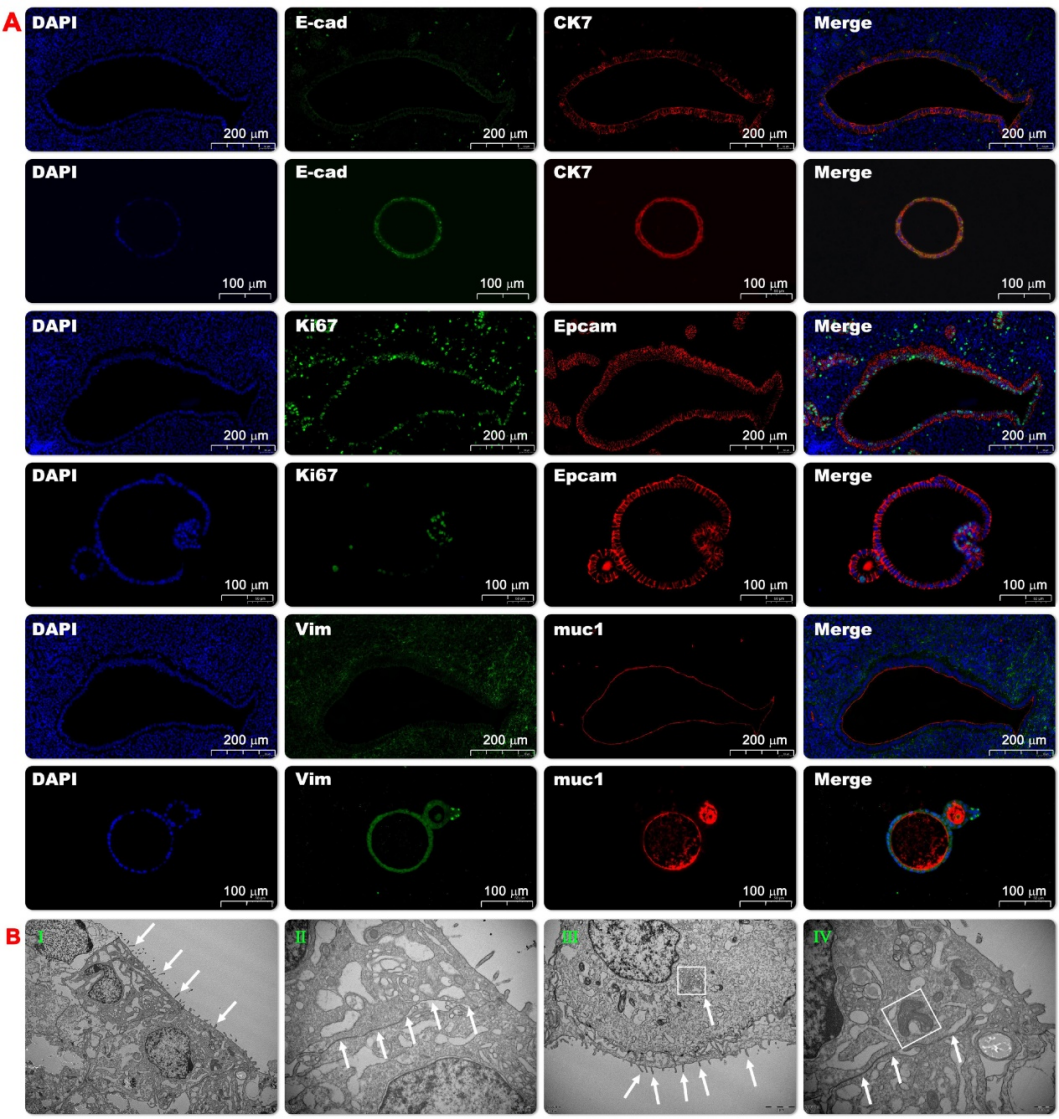
** Morphological identification of endometrial organoids.** The representative pictures of immunofluorescence staining of the co-expression of CK7 and E-cad, Ki67 and EpCAM, Vimentin and muc-1 in the endometrial organoids (20x) and its parental tissue (Fig. 2A). Ultrastructural observation of organoids. Transmission electron microscopy showed that the tubular structure of endometrial organoids was pseudostratified columnar epithelial structure, without lumen or small lumen, and formed apical microvilli (Fig. 2B-I). There were tight connections between the nuclei (Fig. 2B-II). The cytoplasm was rich in rough endoplasmic reticulum and Golgi complex, and there were a large number of secretory vesicles (Fig. 2B-III). In addition, the nucleus was located at the base of the cell, The mitochondrial structure was clearly visible (Fig.2B-IV).

To further clarify the consistence between EMO and its parental tissue, the expression patterns of diverse marker proteins including (secretory) epithelial marker proteins and proliferation proteins were analyzed. High expression level and similar expression pattern of the structure and epithelial markers were observed in EMO as compare to its parental tissue (Figure 1D and 2A). This observation indicates that EMO was derived from the epithelial cell line of the endometrial tissue and it recapitulated the structural feature of the tissue. In addition, the observed expression of MUC-1 on the lumen surface of EMO and columnar epithelium cell appearance suggests that EMOs may maintain the secretory ability of the endometrial glands. The periodic acid—Schiff (PAS) staining showing the presence of the secreted glycogen in the lumen of EMOs further supported this hypothesis (Figure 1C). The presence of Ki67 and SOX9 expressing cells within EMOs indicate the fast-proliferating capability and stemness feature of this organoids model^24^. Taken together, we have established a fast-proliferating EMO model that exhibits endometrial (glandular) epithelial and stem cell phenotypical features and is capable to secrete glycogen. Thus, the appearances are highly similar to endometrial glands in *vivo.* Comparisons were made between mouse organoid cultures established between early passage (p) (2-4p) and late culture (10-15p), and no histological differences were found.

To further illustrate the inner structure of endometrial organoid structures, transmission electron microscopy (TEM) was used to evaluate the organoids structure on days 10 and 14. After taking pictures, virtual slides using tiles of adjacent TEM fields were made to provide a broad field of vision with nano resolution. The wide-field nano-microscope pictures demonstrated that the tubular structure of endometrial organoids was a pseudostratified columnar epithelial structure without lumen (or small lumen), and there was evidence of the formation of apical microvilli (Figure 2B-I). The nucleus was located at the base of the cell and the basement membrane was kept intact. A tight connection was observed between cells (Figure 2B-II). The cytoplasm was rich in rough endoplasmic reticulum and Golgi complexes, and there were a large number of secretory vesicles (Fig. 2BIII and IV). Taken together, we have shown that we could provide detail structures of endometrial organoids using transmission electron microscopy.

### EMOs Showed Physiological and Periodic Simulated Responses to Sex Hormones

**Figure 3:**
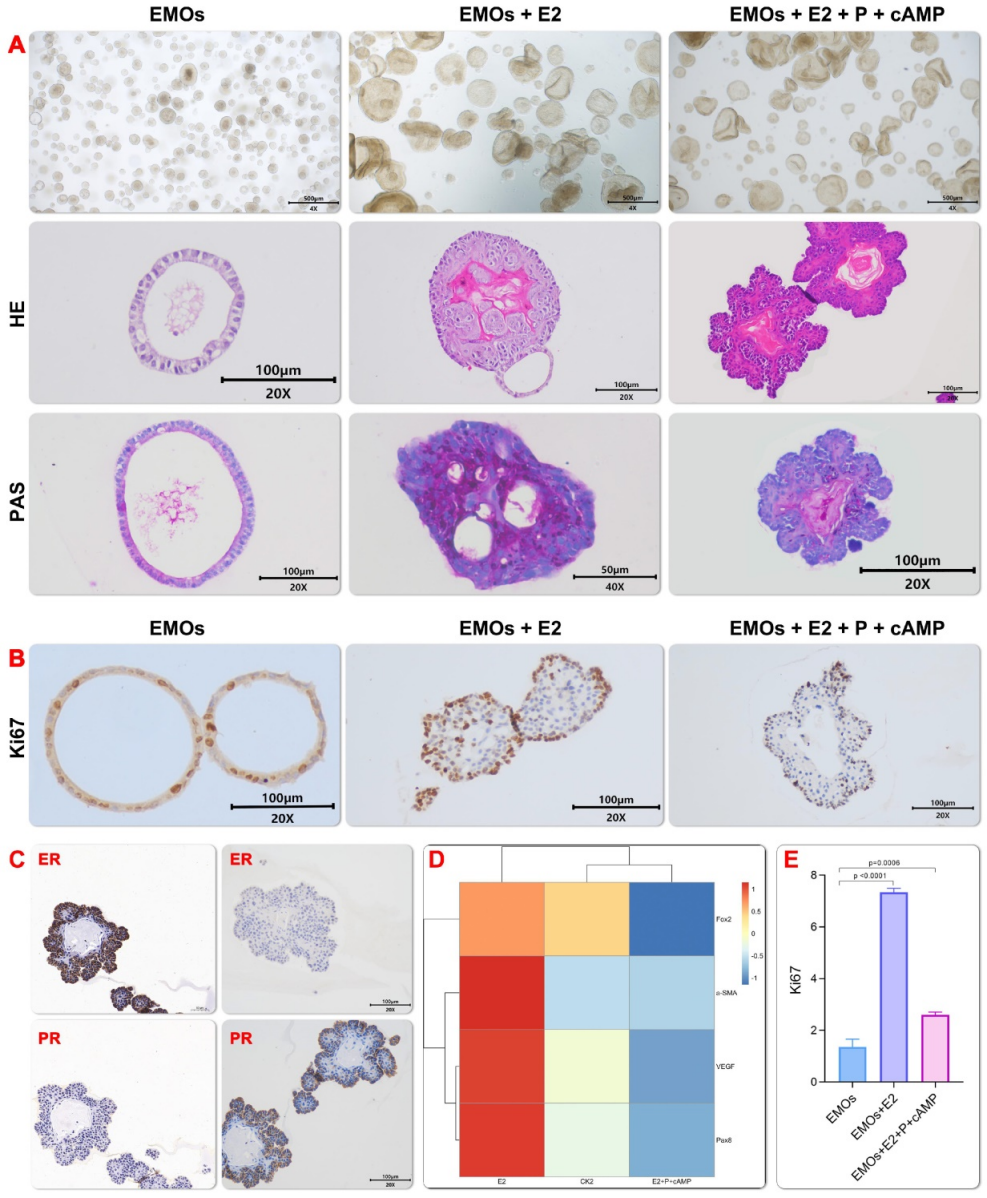
** Endometrial organoids respond to sex hormones.** progesterone treatment simulated the characteristics of endometrial maturation during secretory phase, especially the increase of gland folding and bending, and columnar cells showed subnuclear vacuoles. E2 treated emo epithelium was similar to pseudostratified glandular epithelium in proliferative phase (Fig. 3A). Immunohistochemistry for Ki67 on organoids under EMO, EMO+E2 and EMO+E2+P4+cAMP, and proliferative and secretory endometrium.(Fig. 3B).Immunohistochemistry for ERα and PR on organoids after hormonal stimulation. In ExM expression of ERα is weak, but some cells are either ERαhigh or Erα negative. After E2 and P4 treatment, levels of ERα and PR are higher(Fig.3C). Real time quantitative PCR showed that mouse genes were involved in the development and function of endometrial glands (Foxa2 α-Presence of Sam and Pax8) (Fig.3D).The expression level of Ki67 after induction by estrogen and progesterone was semi-quantitatively analyzed by the area of positive staining.(Fig.3E)

During the menstrual cycle, the change of the estrogen (E2) and progesterone (P4) level will significantly change the proliferation and differentiation status of the endometrium^18^. In order to examine whether generated EMOs could recapture the change of endometrium during the menstrual cycle, especially in the proliferative and secretory stages, the menstrual cycle were reconstructed by adding E2 and subsequently, progesterone P4 to the EMOs culture. An increased expression and decreased expression of Ki67 were observed after addition of the estrogen and progesterone (Figure 3B and 3E), respectively. This finding may indicate that the estrogen treatment induced the proliferation stage during the menstrual cycle, while the progesterone treatment promoted the secretory function of EMOs. The latter finding is consistent with the result of the PAS staining (Figure 3A). In addition, in line with the finding of Turco et al.^18^, E2-treated EMO epithelium was similar to pseudostratified glandular epithelium in the proliferative phase (Figure 3A and 3C). The progesterone treatment led to morphology changes of increased gland folding and bending, which resulted in subnuclear vacuoles formation. Moreover, real-time quantitative PCR showed that murine genes involved in endometrial glandular development and function (Foxa2, α-Sam and Pax8) also emerged (Figure 3D), PAX8+ cells are responsible for long-term maintenance of endometrial epithelium^25^, Foxa2 has been shown to be expressed in the glandular epithelium but not the luminal epithelium, stroma, or myometrium of the mouse uterus^26^.

### Endometrial Organoids (EMO) have Vascularization Function after Transplantation *in vivo*

**Figure 4:**
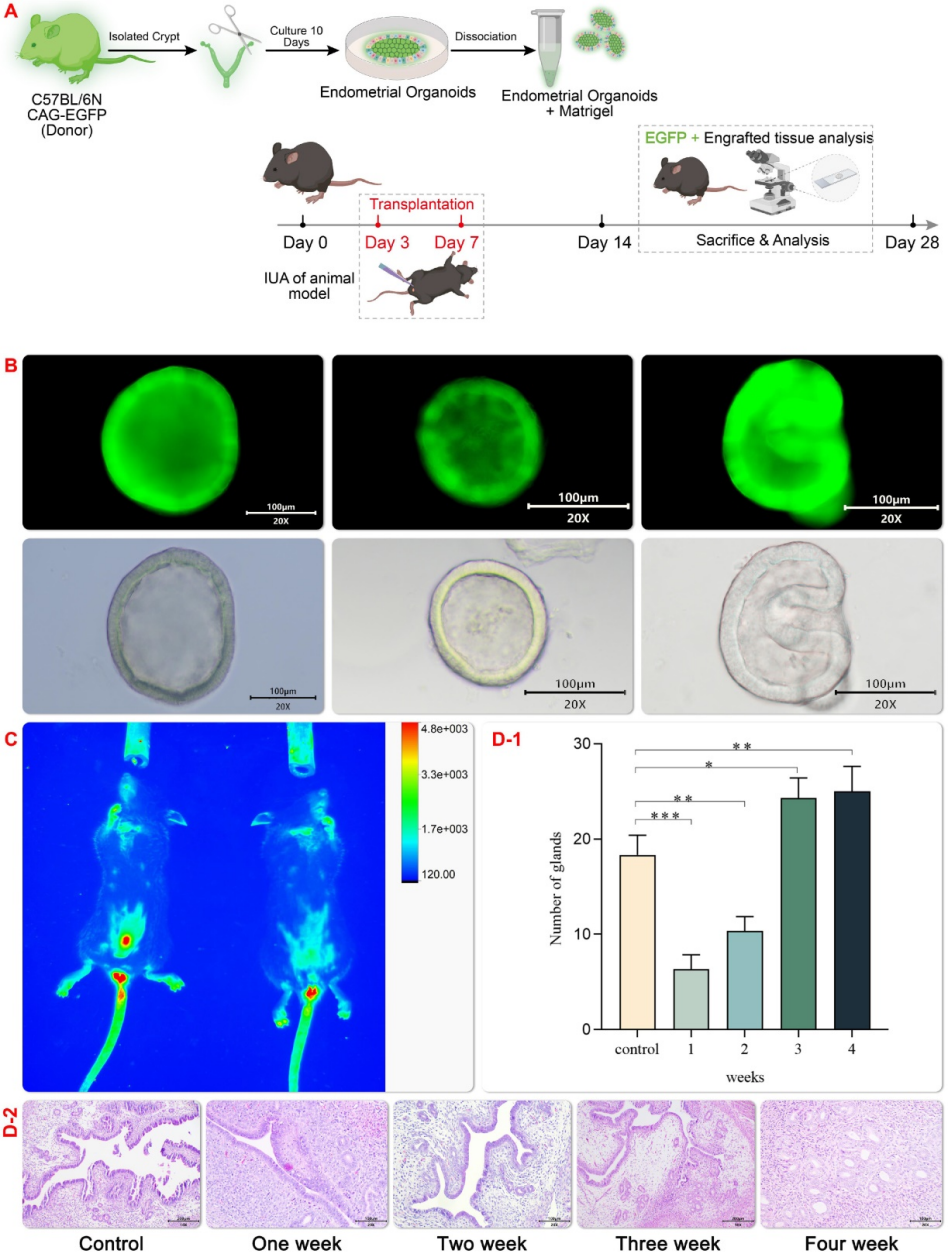
** Establishment of IUA Model and Transplantation of EMOs.** GFP organoids transplantation (Fig. 4A) and schematic diagram of GFP mouse organoids culture (Fig. 4B); Expression pattern of asyn GFP in uterus after homozygous (sncagfp /GFP) organ transplantation. Endogenous GFP fluorescence (red) is displayed(Fig. 4C); After organoids transplantation for uterine injury, uterine horns were collected and stained with hematoxylin and eosin (H & amp; amp; E) (Fig. 4D1-2);4D-2 Endometrial morphology was observed by hematoxylin staining (magnification 20×, scale bar 100 μm). 4D-1 Statistical comparison of endometrial glands in the visual field of each group.Data are reported as means±SD. *P<0.05, **P<0.01.

In this study, EMO transplantation was test to its tissue damage repair effect in the mechanical injury induced IUA model and a schematic experimental flow was shown in Figure 4 A. C57 female mice with a normal estrous cycle of 6-8 weeks were selected to setup the mechanical injury induced IUA model under anesthesia. After repeated mechanical curettage, the diameter of the uterine cavity decreased, the uterine cavity narrowed, and the number of endometrial vascular glands decreased. H&E (hematoxylin-eosin staining) staining showed extensive distribution of the damaged endometrium and disordered proliferation of dark blue collagen fibers (Figure 4D-2). In order to accesses the effect of EMO transplantation *in vivo*, a green fluorescent EMO was generated to track the EMO *in vivo* after transplantation (Figure 4B and C). Hydrogel encapsulated organoid can promote the regeneration and growth of endometrium in mice with uterine injury^27^. In line with this finding, In vivo fluorescence imaging result showed that hydrogel scaffolds can retain the organoid in the injured site for a longer period, probably due to its high temperature sensitivity (Figure 4C). In line with this observation, the expression of several genes including high growth factor (VEGF) was found in the regenerative endometrium near the transplant site. These results show that organoid transplantation provides an encouraging choice for clinical IUA treatment. However, 4 weeks after transplantation, the glands of the transplantation group were significantly higher than those in the control group (Figure 4D-1).

### Fertality Assessment

**Figure 5:**
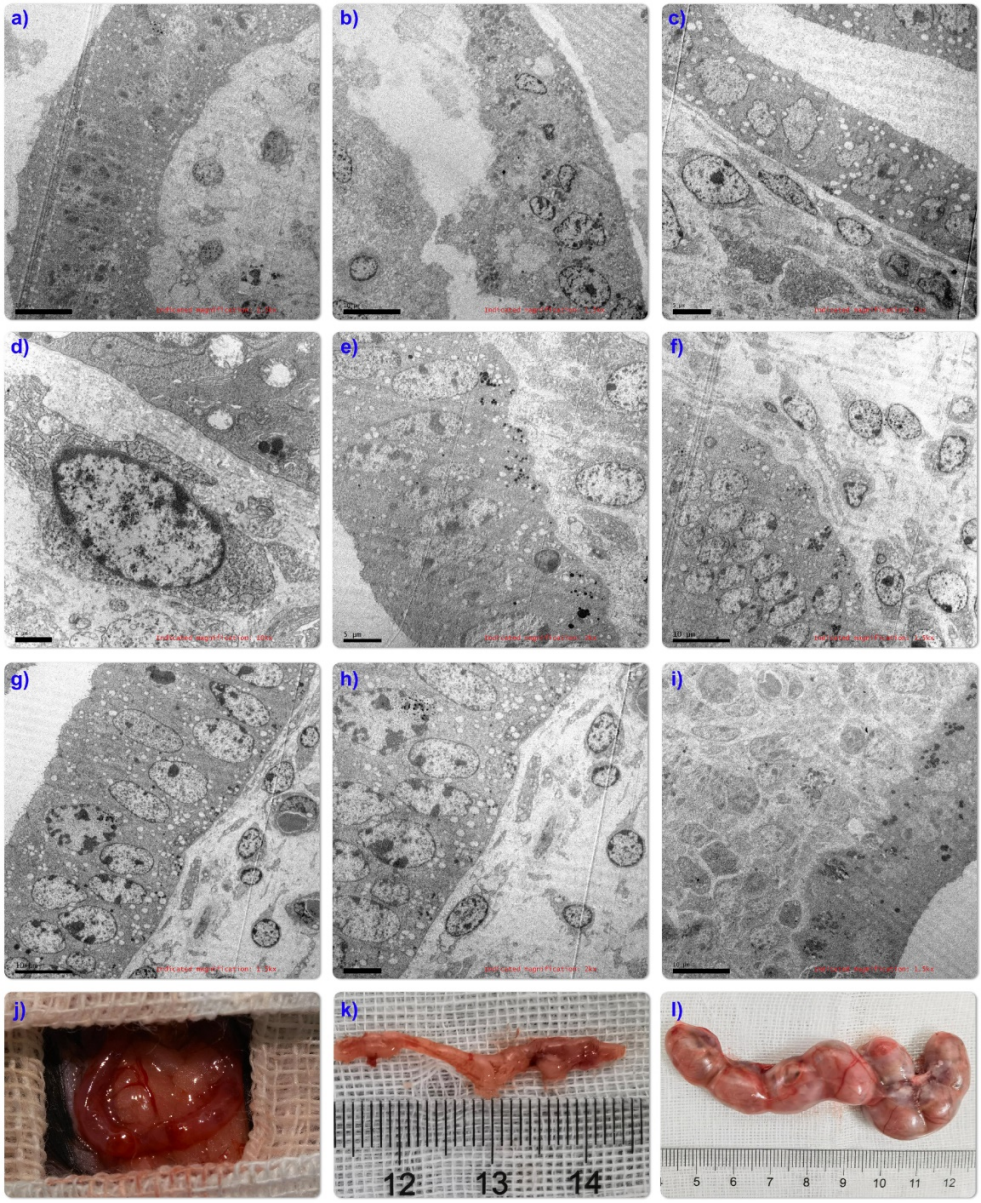
** Ultrastructural evaluation showed that endometrial organoids matured in vitro over time.** Ultra-structure of endometrium in normal mice (Fig. 5A); The results of mouse IUA injury model showed that basal layer defect (Fig. 5B), glandular structure disappeared (Fig. 5C), nucleus lost morphology and a large number of lysosomes were released (Fig.5D); Low-magnifification electron microscopy (TEM) tile scan of endometrial organoids transplanted for 14 days showed the structural interface of columnar epithelium of glands (Fig.5E-J). Exposing the bicornate uterus of C57 mice (Fig.5J); Fertility evaluation experiment found that compared with IUA model group (Fig.5K), organoid transplantation was helpful to improve the pregnancy rate (Fig.5L).

The ultrastructure after organoid transplantation showed that compared with normal mouse uterus (Figure 5A), mechanical damage caused defects in mouse endometrium and muscle layer (Figure 5B). After transplantation, organoid cells spread along the basement membrane and inside the nucleus. Containing a large number of lysosomes (Figure 5C-D), the tubular structures in the transplanted organoids also mature over time, resulting in the polarization of the epithelium into a single layer, and the formation of a developed brush-like top edge area (Figure 5E-H), mature organoids The extensive evidence is very promising. Unfortunately, the arrangement of the organoid structure is uneven, and it has not yet reached the structural state of the orderly arrangement of the glandular tissue in the body (Figure 5I), which may be caused by the difference between the circulation of the organoids outside the body. Therefore, it is necessary to further observe and optimize the experimental device.

In order to detect the effect of emo transplantation on fertility, endometrium of mechanically injured mice (Figure 5J), 4 organoid treated mice and 4 untreated mice were mated with healthy fertile male mice in the ratio of 2:1, respectively. The date of vaginal obstruction was designated as day 0 of pregnancy. On the 15th day of pregnancy, female mice were euthanized. The embryos in the uterine cavity were counted and photographed. IUA model mice treated with EMO transplantations 3/4 were successfully pregnant (Figure 5L), while model mice without emo transplantation were not pregnant (Figure 5K).

### Endometrial Organoids (EMO) Mature Gradually

**Figure 6:**
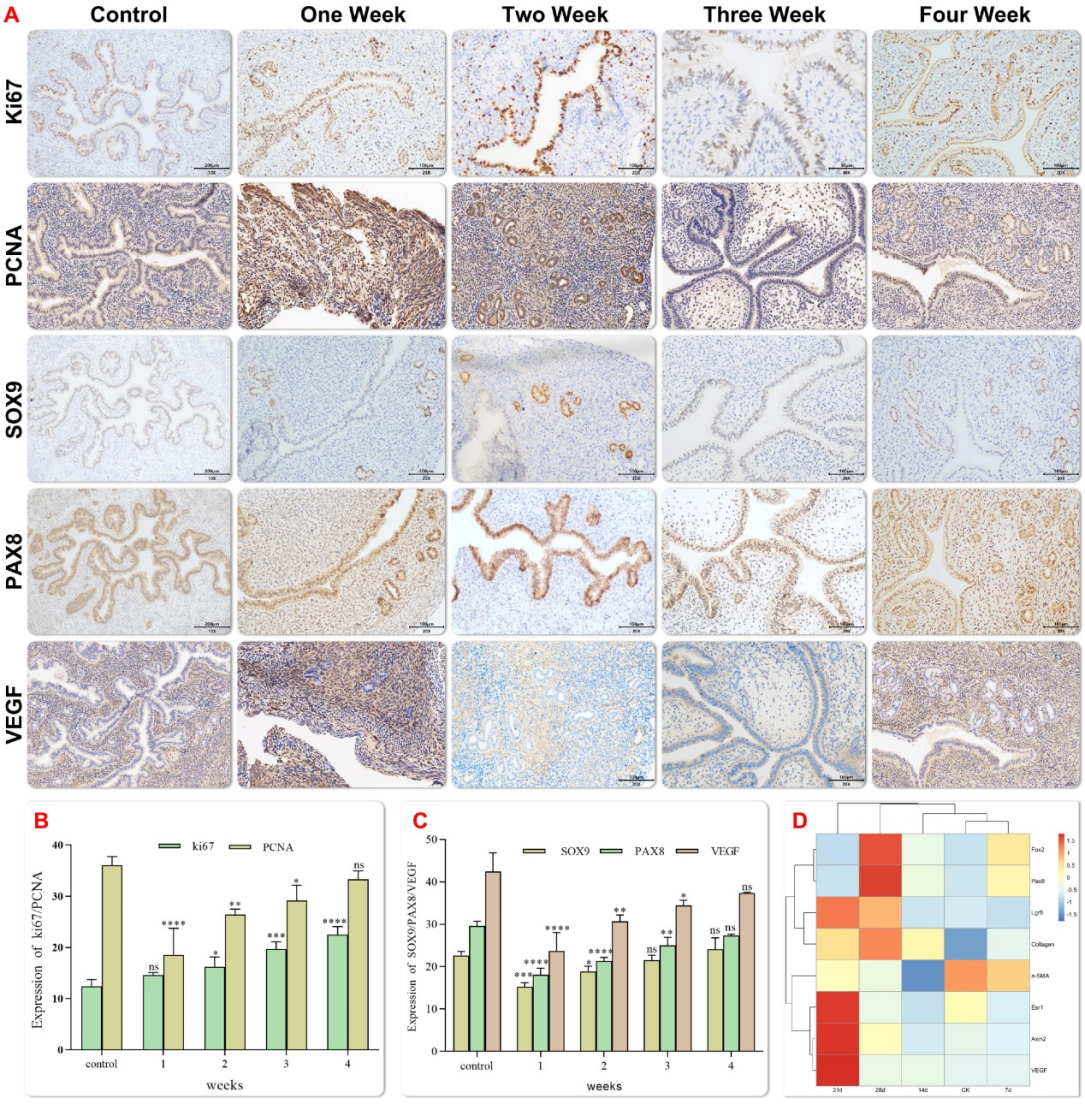
** Histology of experimental Asherman syndrome in mice.** Immunohistochemical staining showed representative images of Ki67, Sox9, Pax8, VEGF and PCNA localization in endometrial tissues of each group(magnification 20×, scale bar 100 μm) (Fig. 6A). The expression levels of Ki67, Sox9, Pax8, VEGF and PCNA were semi quantified by positive staining area. The data were reported as mean ± standard deviation * P < 0.05, * * P < 0.01 (Fig. 6B-C). Gene expression was analyzed by Q-PCR at each time after organoid transplantation (Fig. 6D).

Vascularization of the graft is a key determinant for successful integration into host tissue. In order to determine the effect of EMO transplantation in the mechanical injury induced IUA model, the expression of the angiogenesis marker (vascular endothelial growth factor, VEGF) and the progenitor cell marker (Sox9) were examined at 1, 2, 3 and 4 weeks after transplantation. Increased trends of expression of both proteins were observed after transplantation over the time (Figure 6A-C).Newly formed endometrial tissue was measured by evaluating the expression of Ki67 in proliferating cells. Ki67 is a nuclear antigen at different stages of cell proliferation, and its expression reflects the regeneration of the damaged endometrium. Immunohistochemical staining showed that Ki67 was expressed in all groups, and the expression of Ki67 was the highest in epithelial cells 4 weeks after transplantation, which confirmed the improvement in the repair efficiency of endometrial injury 4 weeks after transplantation (Figure 6A and B). Immunohistochemical and qPCR analysis showed that the expression of VEGF and adipocyte-specific adhesion factor increased significantly after transplantation, (Figure 6D). The expression of SMA and other growth factors was up-regulated. Additionally, the deposition of the extracellular matrix (ECM) is associated with the down-regulation of COL1A1 expression after transplantation. Fourteen days after the transplantation, the results of projection electron microscopy showed that porous special endothelial structures appeared in organoid, and tight junctions migrated from the top to the bottom. These observations were in line with the previous findings of podocyte maturation during glomerular capillary ring formation^28^.

## Discussion

A reliable endometrial model is the key to better understand the interaction between endometrial cells and the environment, which is also an important tool to study endometrial related diseases. In this study, we have demonstrated that endometrial organoids (EMO) recapitulate the morphology and molecular change of the function lining of endometrium *in vitro* and could promote the tissue repair in a tissue damage induced IUA mice model.

In contrast to 2D primary stem cell culture, EMO has been shown to exhibits the phenotypical characteristics of stem cells and could differentiate into multiple cell types. In this study, we have generated the EMO from the proliferative uterus of 6-8-week-old mice using a EMO specific culture medium updated from previous report^14^. We have found that the generated EMO composed of ciliated luminal epithelial cells and secretory glandular epithelial cell. Moreover, a detailed morphology of these cells such as pseudostratified columnar epithelium without cavity (or small cavity) and the apical microvilli were demonstrated using electron microscope. These findings were in line with previous reports that demonstrated the organoids models could represent the key structural features of its original tissue^9^, ^29^-^32^. In addition, exposing EMOs periodic to sex hormones, we have shown that the generated EMOs could recapitulate the morphology changes and physiological status of the endometrium during the menstrual cycle. Taken together, in line with previous reports we have generated an *ex-vivo* EMO model that exhibits the phenotypical characteristics of the primary endometrium tissue and can recapitulate the function of the original tissue.

In this study, we have setup a mouse IUA model to access the tissue repair effect of EMOs. Although it is difficult to monitor the endometrial thickness of mice, reproductive outcome is the best proof of whether the endometrium recovered from the tissue damage both on morphology level and functional level. Rescued reproduce capability of the mice after EMO transplantation indicate that EMO transplantation promotes the regeneration ability of endometrium. In addition, increased host blood vessels penetration and the expression level of vascular growth factors (including VEGF) in endometrial progenitor cells of the grafts were found in mice two weeks after EMO transplantation. This observation suggests that the EMO transplantation promote the tissue repair by improving the vascularization among the damaged tissue, although the precise mechanisms still need to be investigated.

In this study, we have mixed the EMOs with a temperature controlled hydrogel prior transplantation^33^, since several cases have been reported that organoids were fragmented after transplantation. After transplantation, the transplantation mix containing EMOs can gradually form a jelly like bio-scaffold in the uterine cavity under the influence of body temperature. It would not oppress the endometrium and affect the blood flow under the endometrium since it becomes soft at body temperature. In addition, its unique properties allow it to release EMOs gradually and it will degrade spontaneously during the release process. Moreover, the hydrogel mix containing EMOs is closely linked to organ wall and preserves the tissue structure of the organ. Summarized, this hydrogel - transplantation mix can protect the donor cells from the harmful microenvironment of the graft and keep the EMO in the injured area for a long time. These unique characteristics make a good potential candidate for treatment against IUA.

## Conclusion

In this study, we have generated an ex-vivo EMO model that represents the key phenotypical- and functional characteristics of the endometrium tissue. In addition, we have shown that transplantation of EMO-hydrogen mix could promotes the tissue repair and rescue the reproduce capability of tissue injury induced IUA in mice. This study is of great significance to clarify the mechanism and safety of organoid transplantation in the treatment of IUA. It not only provides a theoretical basis for clinical application, but also focuses on the safety of organoids, application methods, and the preparation of related biological products. As such, we believe that organoids transplantation could be a potential tool that could support the treatment of IUA.
